# Beyond Rhythm Recognition: A Clinical Pathway for Smartwatch-Detected Atrial Fibrillation

**DOI:** 10.1016/j.jacadv.2026.103300

**Published:** 2026-10-06

**Authors:** Yingying Wang, Wanhaier Abudoureheman, Qiuzi Sun

**Affiliations:** a Department of Cardiology, Longhua Hospital Shanghai University of Traditional Chinese Medicine, Shanghai, China; b Department of Nephrology, Longhua Hospital Shanghai University of Traditional Chinese Medicine, Shanghai, China; c Department of Nursing, Longhua Hospital Shanghai University of Traditional Chinese Medicine, Shanghai, China

Avidan et al^1^ conclude that primary care physicians are insufficiently equipped to interpret smartwatch electrocardiograms (ECGs) and require targeted training. We believe this framing risks equating rhythm-label accuracy with clinical competence. In primary care, the safest response to a consumer-generated single-lead tracing is not always to name the rhythm; it may be to prevent an isolated, potentially misleading recording from driving treatment.

Particularly concerning is the implied progression from smartwatch misinterpretation to inappropriate anticoagulation. Current guidance requires clinician review of the electrocardiographic tracing to establish atrial fibrillation (AF), while anticoagulation depends on individualized thromboembolic and bleeding risks rather than a device label.^2^ Even after a device-detected atrial arrhythmia is verified, treatment benefit is not automatic. NOAH-AFNET 6 found that edoxaban did not reduce the primary efficacy outcome but increased the composite of death or major bleeding,^3^ whereas ARTESiA showed fewer strokes or systemic emboli with apixaban at the cost of more major bleeding.^4^ Although these trials involved implanted-device-detected subclinical AF, they expose the clinical error of collapsing “AF detected” into “anticoagulate.” Training focused mainly on label agreement could therefore increase confidence without improving decisions.

The converse problem is equally important. A normal 30-second smartwatch ECG cannot exclude paroxysmal AF; intermittent sampling has low sensitivity relative to continuous monitoring.^5^ A primary care physician who accepts “sinus rhythm” and reassures a persistently symptomatic patient may be less safe than one who acknowledges uncertainty and seeks specialist review.

Training should therefore prioritize an escalation pathway: identify symptoms requiring urgent assessment, obtain confirmatory 12-lead or ambulatory ECG monitoring, evaluate alternative arrhythmias, and only after AF is verified, individualize stroke-prevention and rhythm-control decisions. The clinically meaningful competency is appropriate action, not concordance with a rhythm label. Without this distinction, the authors’ conclusion risks encouraging decisiveness precisely where caution is safer.
